# Handling polypharmacy –a qualitative study using focus group interviews with older patients, their relatives, and healthcare professionals

**DOI:** 10.1186/s12877-023-04131-6

**Published:** 2023-08-08

**Authors:** Thorbjørn Hougaard Mikkelsen, Jens Søndergaard, Niels Kristian Kjaer, Jesper Bo Nielsen, Jesper Ryg, Lene Juel Kjeldsen, Christian Backer Mogensen

**Affiliations:** 1 Emergency Department, Hospital Sønderjylland, Aabenraa, Denmark; 2 Research Unit of Emergency Medicine, Department of Regional Health Research, University of Southern Denmark, Odense, Denmark; 3 Research Unit of General Practice, Department of Public Health, University of Southern Denmark, Odense, Denmark; 4 Department of Clinical Research, University of Southern Denmark, Odense, Denmark; 5 Department of Geriatric Medicine, Odense University Hospital, 6 The, Odense, Denmark; 6 Hospital Sønderjylland, Kresten Philipsens vej 15, indgang F, Aabenraa, 6200 Denmark

**Keywords:** Polypharmacy, Older people, Non-adherence, Adverse drug interactions, Medication errors, Experiences, Continuity of care, Focus group interviews, Qualitative research

## Abstract

**Background:**

On average, older patients use five or more medications daily. A consequence is an increased risk of adverse drug reactions, interactions, or medication errors. Therefore, it is important to understand the challenges experienced by the patients, relatives, and healthcare professionals pertinent to the concomitant use of many drugs.

**Methods:**

We conducted a qualitative study using focus group interviews to collect information from patients, relatives, and healthcare professionals regarding older patients’ management of prescribed medicine. We interviewed seven patients using five or more medications daily, three relatives, three general practitioners, nine nurses from different healthcare sectors, one home care assistant, two hospital physicians, and four pharmacists.

**Results:**

The following themes were identified: (1) Unintentional non-adherence, (2) Intentional non-adherence, (3) Generic substitution, (4) Medication lists, (5) Timing and medication schedule, (6) Medication reviews and (7) Dose dispensing/pill organizers.

**Conclusion:**

Medication is the subject of concern among patients and relatives. They become confused and insecure about information from different actors and the package leaflets. Therefore, patients often request a thorough medication review to provide an overview, knowledge of possible side effects and interactions, and a clarification of the medication’s timing. In addition, patients, relatives and nurses all request an indication of when medicine should be taken, including allowable deviations from this timing. Therefore, prescribing physicians should prioritize communicating information regarding these matters when prescribing.

**Supplementary Information:**

The online version contains supplementary material available at 10.1186/s12877-023-04131-6.

## Background

Generally, senior citizens use five or more prescription medications daily [1]. While an individual prescription solves a problem, the combined medications do not always give the expected result [2–7], with some patients experiencing severe side effects [8–12]. In addition, polypharmacy increases the risk of drug-drug interactions, especially among older patients [13, 14].

Healthcare sector transitions increase the risk of information loss, misunderstandings, unclear treatment responsibilities, and medication errors [15–17]. Medication management of the older patient after hospital discharge is a complex process [18]. The complexity depends on the number of medications, frequency and length of time medications should be taken, frequency of sector changes and the number of health professionals involved, the patient’s and occasionally relatives’ cognitive function, as well as the patient’s motivation to take the medication [19].

In Denmark, all patient-prescribed medications are listed in the “Fælles Medicinkort”, an online Shared Medication Record (SMR) accessible by patients and healthcare professionals across sectors [20–22].

The patients are central to ensuring correct medication, as they are often responsible for buying, remembering and taking their medication [23]. One Danish study reported that 7.5% of patients aged 70 + years fail to redeem the first prescription of a new drug prescribed in general practice [24].

Research shows that patients experience medication errors, misunderstandings, discontinuity, information loss, and other obstacles after sector transitions [15–17, 25]. Understanding the issues experienced by patients and their relatives is crucial for improving adherence. However, as medication treatments are initiated and monitored by healthcare professionals, including pharmacists, nurses, and physicians, it is crucial to triangulate the experiences to become aware of other relevant aspects.

### Aim

This study explored the medication challenges that older patients treated with five or more drugs met.

## Methods

### Setting and participants

We performed qualitative semi-structured focus group interviews (FGI’s) among patients, relatives, and healthcare professionals to represent experiences from various central actors. FGI’s are beneficial in exploring an informant’s knowledge and perspectives [26]. In addition, we chose FGI’s to obtain synergetic effects and encourage informants to elaborate on the challenges and practices of managing their medication [26–28]. The interview guides (translated from Danish) are attached as supplementary file 1.

### Interviews

Inclusion was based on consecutive sampling among patients admitted to the Emergency Department at Hospital Sønderjylland for ten days in April, May, and June 2021. In addition, the healthcare professionals were included by purposeful sampling inviting specific people from the primary and secondary healthcare sectors from the same uptake area.

Patients admitted acutely to the emergency department were invited to participate if they were 72 years or older and managed five or more medications themselves or with the help of a relative. The first author identified patients that matched the age inclusion criteria and excluded patients with known dementia. Then, the last author assessed if the patient met the inclusion criteria, e.g. examined how many medications the patient took daily and contacted the nurse responsible for the patient to confirm that the patient matched the inclusion criteria. Next, the nurse asked the patient if the first author (THM) could contact them regarding the research project. If the patients accepted, THM approached them, informed them, and invited them to participate. If the patient agreed to participate, the interviewer (THM) informed them in more detail verbally and in writing about the details of the study.

Patients who agreed to participate were invited to participate in a focus group interview with other patients at a later date. Overall 31 patients were eligible. One patient were not invited due to confusion. In addition some patients were either discharged or transferred to another department before they were contacted. A total of 10 patients, here of three with a spouse, accepted the invitation to the FGIs. One of these died before the FGI and another did not show up. In the FGI with the relatives’ one of the patients were too ill to participate while the spouse participated.

Hospital staff were invited by an internal email sent to the relevant departments. An email was sent to head nurses at the departments of geriatric, internal medicine, and emergency at Hospital Sønderjylland, asking if they could contribute to the study by allowing one of their nurses to participate. Consultants from the same departments were invited directly by email. Hospital and local pharmacists were also invited to participate directly by mail. Homecare nurses were invited via an email to the municipalities in the uptake area. General practitioners (GPs) and nurses employed in general practice were invited by an email to the general practices familiar to the authors and also welcomed other colleagues from the uptake area.

At startup, the informants were informed verbally and in writing about the study’s details and asked to sign a consent form.

Five FGI were conducted with various healthcare professionals, and in FGI E a patient and a relative were also included:

- FGI A − 2 chief physicians (geriatric and internal medicine), 1 hospital nurse (Emergency Department), and 1 hospital-employed pharmacist.
- FGI B − 5 nurses from general practice.
- FGI C − 2 homecare nurses, 1 homecare assistant, and 2 pharmacists.
- FGI D − 2 GPs.
- FGI E − 1 GP, 1 homecare nurse, 1 hospital-employed pharmacist, 1 patient, and 1 relative.

Three FGI were conducted with patients as well as patients and relatives:

- FGI F − 2 patients and 3 relatives.
- FGI G − 2 Patients.
- FGI H − 3 patients.

If possible, we wanted to carry out an FGI with health professionals, patients, and relatives. Hence the patient and the relative in FGI E also participated in FGI F with patients and relatives. Furthermore, the patient and relative were invited to FGI E after participation in FGI F, because they could enrich the dialogue by interacting with the healthcare professionals (see Table 1 for patient and relatives characteristics).

In total, 29 informants with different backgrounds participated.

**Table 1:** Characteristics of the participating patients and relatives

Focus group interview	Patient alias	Age of the patient	Participating relatives
FGI F with both patients and relatives			
Male	M	74	Relative = K
Male	Y*	78	Relative = D*
Nonparticipating male patient, relative attended		73	Relative = X
Patient FGI G			
Male 1	A	79	
Male 2	B	77	
Patient FGI H			
Female 1	C	83	
Female 2	D	75	
Female 3	E	73	

* Also participated in the FGI E with a GP, pharmacist, and home care nurse

### Data collection

All interviews were conducted at the hospital, except for FGI B and D, which took place at the local GP’s clinic. The first author completed all focus groups using a semi-structured interview guide targeting the specific focus group and using open-ended prompt format [26, 29]. See the semi-structured interview guides in the supplementary files.

### Data analysis

Interviews were transcribed as well as coded, sorted, and managed in Nvivo [30]. Recruitment to the study aimed at achieving rich and diverse perspectives as well as varied and sufficiently large to elucidate our aim [31].

The beginning of all interviews were framed with the following introduction:

”The purpose of this project is to develop a method or solution so that patients receive the correct medication after discharge from hospital focusing on patients over 71 years old, prescribed five or more types of medication after discharge”.

Initially all interviews were read to obtain an overview over the material before coding. The text was read a second time to identify the meaning units. We applied an inductive approach focusing on the informants’ perceptions, understandings, and ideas. We also applied a deductive analytic strategy based on the themes of the interview guide. Hence we coded the interviews after the themes in the interview guide as well as emerging themes. The group discussions were analyzed phenomenally, focusing on the informants’ experiences and perceptions of things and events [29, 31]. Based on the coded data material we created condensed themes. Quotes are used illustratively.

The research team contributed with their knowledge and expertise: THM is a sociologist, JS a clinical pharmacologist and GP, NK a GP, JBN is an expert in communication, JR a chief physician in geriatric medicines, LJK is a trained pharmacist and CBM is a chief physician in internal medicine.

## Results

Issues identified through the FGIs fell into seven categories: (1) Unintentional non-adherence, (2) Intentional non-adherence, (3) Generic substitution, (4) Medication lists, (5) Timing and medication schedule, (6) Medication reviews and (7) Dose dispensing/Pill organizers.

### Unintentional non-adherence

In general, patients and relatives do not experience problems remembering their medicine, but if they are not in their home environment or are focused on something else, medication can sometimes be forgotten. One strategy used was a telephone alarm as a reminder:

*Relative D: Remembering medication, we have an alarm on our phone because we have a vegetable garden, and when you walk around in the garden, you forget what time it is, so I always have an alarm reminder.*

Disruptions of routines, for example, during holidays, can cause forgetfulness:

*Relative X: Yes, that’s what I’m thinking about too. What you can forget about it is that if you are on holiday in a hotel and you have to eat but have forgotten your pills. You have to remember to have them with you every time.*

These experiences match healthcare professionals’ experiences that without a routine, patients often forget, e.g. a penicillin regimen: A routine support to remember to take the medication is useful. Therefore, younger and more active people primarily benefit from medication reminders on the telephone.

*Pharmacist Y:… and it’s not that easy. I just had a course of penicillin for 8 days, and again and again I forgot it, I’m at work in the morning, I forgot it. In the afternoon I was busy, I forgot it again, so I only had one tablet all day. It happens, it’s really hard to remember. “God, I forgot it again”, and I give advice every single day, every single second behind the counter, “remember to take your medication” right?*

There is a big difference in how patients manage their medication. Some store the medication in a bag they take out as needed, others distribute them into small containers the night before, and some store them in the kitchen cupboard. Interestingly none of the informants used a telephone app to manage medications.

Many women manage their own medication, whilst men, tend to receive help from a partner:

*Patient M: My wife controls my medication because we have those pillboxes.*

*Relative K: Well, every Friday it must be refilled. And I sit down and put the medication in the right boxes. Then in the morning, he gets it (the pillbox), swallows the pills when he eats.*

### Intentional non-adherence

Some of the patients are intentionally non-compliant and one patient decided not to take the newly prescribed medication after reading the package leaflet:

*Relative D: Yes, my husband, needed to take a new kind (of medication) and he also has a bad kidney, and then he read that kidney patients should not take them. So that’s why he didn’t take them. And so when we came to the hospital for control, we told them. Then they said, well such a small dose, you can tolerate, it’s only 4mg or something like that. But still, he damn well did not want them. But he had to do it anyway, because it helped the kidney test results, after all.*

As one patient explained:

*Patient Y: …when I read the leaflet I was thinking, oh my God, I can get a bowel obstruction or constipation. Then I put it up on the shelf because I’d had one before (bowel obstruction), I knew I wouldn’t dare, whilst I was on so much medication, I thought they might affect each other negatively. I had to tell them that I hadn’t taken this powder, but I kept to the diet in another way. It achieves the same.*

After reading the package leaflet, the patients were concerned about the side effects and therefore chose to avoid taking the medication.

Another patient described that after a minor operation, he stopped using a new painkiller (morphine) after experiencing uncomfortable side effects such as vomiting and chose to use paracetamol and accept a degree of pain. However, other patients seek professional advice before altering their medication.

*Patient Y: … when I was taking a diuretic, I said that I knew the dose was too high - and after I told them, they said, OK let’s reduce the dosage now.*

Thus, intentionally choosing non-compliance is a phenomenon relatively common among patients.

### Generic substitution

One patient explains that dealing with polypharmacy in everyday life is difficult. She describes an ingenious system in her kitchen cupboard, where the medication boxes are arranged in a row with names, numbers, and times written on shelf stickers under the boxes:

*Patient E: About 7 pills in the morning and 4 or 5 at night. It is written there on the shelf, just like in the pharmacy. But that’s also the problem, the moment you come to the pharmacy, they change the boxes. Then you have to change your whole system of rows. There are a lot of copy products and it’s rare to get the same original pills. They are often out of stock when you come in. Then you get some new ones- so you have to change… and the labelling on the packet says nothing… It has been really troublesome. I think it’s really difficult.*

This patient had been through many assessments at several hospitals and many changes in medication. If the prescribed medication is out of stock, she often has to buy a generic product resulting in a different brand, name, and packing, which is confusing for the patient. This was described well by one of the general practitioners:

*General practitioner L: It is a big challenge…from the general practice perspective. There is a constant shift between medical brands because of the price, and the pharmacy is obliged to take the cheaper product. The medication then looks different, the packaging is different, and it does confuse older people extremely.*

### Medication lists

A treating physician or pharmacist often gives patients a medication list. It is a great help for the patient and enables them to track their medication. However, it can confuse patients when the medication has a name that does not correspond with the patient’s medication list. For example, a nurse from general practice explains:

*Primary Care Nurse 4: It’s quite a cocktail they sometimes come up with. And some become confused, but I think it’s more due to the names. I think the hospital is good at printing medication lists and giving the patient the medication (to ensure they have enough until they manage to get to a pharmacy (ed)). I actually think most people get it. Nevertheless, the problem is that it is just not called the same.*

A general practitioner also acknowledges the problem and suggests an addition to the list:

*General practitioner 2: Maybe you should write the generic and alternative names on the list of medications that’s handed out to the patients because it’s definitely a huge problem.*

This simple, practical solution may reduce the patients’ confusion when they are given copy medication instead of the prescribed medication, which is a problem that preoccupies and confuses patients.

### Timing and medication schedule

Many patients expressed doubt about the timing of the medication intake when instructions indicate 3 or 4 times a day.

*Relative K: You aren’t really told how long the time should be in-between. At the moment, he gets quite a lot of medication in the morning, lunch is around 12 − 1 o’clock, but when he gets these lunch tablets does that harmonize with the fact that it is so soon after he has received his morning tablets? Because M, you like to sleep until 9–9: 30, so lunch is between 12 − 1. So we asked if it was OK? That’s good enough, they said… I just think...*

As the quote shows, morning is not a straightforward concept, and when the patient gets up late, there is a relatively short time between morning and lunch medication. Therefore, patients and relatives would like more precise statements about how the medication is best taken, even if it only regards one pill per day in combination with other kinds of medication:

*Relative D: Now I have also brought my husband’s medication list with me. The first page says morning, noon, evening, and night, and that’s fine - then I know when he should have them. But then further down there is just one capsule daily, and another that does not say when… it just says the dosage and nothing else.*

Patients need to be given a recommended timetable when taking multiple prescriptions to relieve anxiety about possible interactions with other medications.

### Medication reviews

The combination of many different doctors, pharmacist alerts to medication interactions, and warnings on package leaflets combined with symptoms results in confusion and insecurity among patients and relatives:

*Relative K:… at the pharmacy, they said that these two medications don’t match together well, and they could perhaps find another one. ” Well, what’s best for you, is what you need. ” (said the doctor, ed.). It’s as if no one wants to go in and revise it. Once you get a diagnosis …., then you get some pills that you keep getting. They just get prescribed all the time. No one goes in and revises… It can be a little frustrating sometimes. Also when you see how bad he gets, because he can’t keep his balance. It’s hard to get a clarification somewhere about it.*

…

*Patient M: And then he (the doctor, ed.) says, “Well, I can’t do that”, or “No, it was (prescribed at ed.) the heart department”. “I can’t deal with that, you’ll have to call the hospital, the cardiac department ”. So, I did. Then the cardiac department says, “Your own doctor could do that.“ So you just get thrown back and forth”.*

Patients with polypharmacy are very concerned about side effects and adverse drug reactions, stemming from combining medications; hence they search for better options for a medical review. However, patients often experience doctors’ reluctance to reconsider or change prescriptions if initiated by other doctors.

Although general practitioners and hospital physicians take responsibility and adhere to the medication list, it is not always sufficient reassurance for the patient:

*Patient M: I asked if taking all those tablets on the medication list was necessary. I asked my own doctor and here in Aabenraa at the hospital. And they said, “Well, these are the tablets you have to take.“*

The fear of medication interactions can weaken patients’ and relatives’ trust in doctors:

*Relative K: It’s a bit annoying. You’re left with a feeling of “argh”. Well, I don’t mean to say that they don’t know what they are doing, but sometimes you think, it’s some kind of “oops” (random ed.) the lot of it.*

In attempting to navigate between many different and divergent, contradictory pieces of information, patients’ and relatives’ trust in information from health professionals can be eroded. Interestingly, all participating patients and relatives expressed a degree of insecurity regarding their medication.

### Dose dispensing/pill organizers

Dose dispensing using blisters delivered from the pharmacy or pill organizers packed by the municipality’s homecare team are options available to the patient to reduce medication errors. A few patients had tried pre-packed pill organizers and indicated they were satisfied with the solution, but none had tried dose dispensing using blisters.

*Patient Y: When I came home from the hospital and had been through extensive heart surgery, they filled me with medication to get me up (on my feet (ed.)) again. We went to the municipality’s homecare team and got them to pack my tablets. And that meant that they packed my medication in pill organizers a month at a time.*

*Interviewer: okay, so they come in small packages? Morning, lunch, night, etc. Right?*

*Patient Y: Yes, morning, lunch, and evening. It works damn well.*

*Relative X: And if there are changes to the medication, they fix that too. Mostly, if it’s just one (pill ed), and I know which pill it is, then I remove It from the pill organizer, but otherwise, the municipality comes and does it.*

Although the patient and his relative are competent, there is an obvious risk of incorrect medication. This risk is present for all patients that manage their own medication but is higher for patients managing multiple prescriptions.

Patients expressed satisfaction with dose packing in pill organizers by the homecare team:

*M (mixed FGI): We are so happy that we have the nurses to pack my medication, it’s worth its weight in gold. It is so important to know the timing of the medication.*

No patients experienced or mentioned the cost of having the medicine packed by the homecare. While no patients had tried dose dispensing using blisters delivered from the pharmacy, this solution was mentioned by healthcare professionals who stated that dose-dispensing using blisters could either delay changes in the medication or be expensive for the patient if the blister-packed medication is thrown away:

*Primary Care Nurse 4: We would rather not use it. When the medication is changed, it takes 14 days before the changes take effect.*

*Chief Physician M: Dose dispensing. … it’s a bloody nuisance, as far as we are concerned, because it is a huge problem when you’re hospitalized, and we need to make (medication, ed) changes. There can be a huge expense associated with it, especially if the patients have just got a new roll (of tablets, ed).*

Thus, healthcare professionals find that dose dispensing using blisters can be expensive if pre-packed medication must be repackaged or thrown away, for example, because of changes to a patient’s medication or may delay adjustments to changes hence risking incorrect medication. However, for patients in contact with the municipality, homecare can be helpful and provide professional packaging of medication in pill organizers free of charge.

## Discussion

This study furthers our understanding of why older patients with polypharmacy continue to have issues regarding medication management and often ask for a medication review. First of all, this study shows that older patients and nurses request an accurate indication of the timing of the medication. Despite all patients’ interest in managing their medication, information describing adverse events and harm from various sources results in confusion and, for some patients, intentional non-adherence. Therefore, many older patients request medication reviews simply for reassurance about correct medication and the opportunity to obtain more explicit guidelines, such as the most optimal time to take the medication, to minimize the risk of interactions. In addition, the pharmacy’s legal obligation to supply the cheapest medication results in constant changes in the brand names of medications contributing to the confusion of patients and many requests for medication reviews. Hence the medication lists handed out to the patients might be improved by adding the names of the generic and alternative medications.

Medication management is a task that must be fitted into daily routines and differs substantially between patients [32]. For example, in this study, some patients stored their medication in a plastic bag whilst others organized it in medication boxes packed for a week or months packed by a relative or supplied by healthcare professionals employed at the municipality.

### Medication lists

Medication lists are important for patients to track what medication they are using. A treating physician or pharmacist often hands out the medication list to the patient. However, it can confuse patients when the medication has a name that does not correspond with the patient’s medication list due to generic substitution.

### Generic substitution

Previous research has shown that patients become confused and uncertain when original-brand name medication is substituted for a cheaper generic medication, increasing the risk of the patient making medication errors [33–36]. This study supported these results with patients and healthcare professionals describing this problem. Patients suggested solutions similar to other studies, such as insisting on the original prescribed medication despite associated higher medication costs [36]. Another solution proposed by a GP was that the overview with the originally prescribed brand was supplemented with the generic products that can replace them. This simple solution could be quickly adapted and matches the older patient’s wishes and needs well. However, such a list may be quite extensive. An alternative could be a pharmacist adding the new generic medication to the patient’s medication list. If the medication list is too extensive, an app could be developed to provide an alternative solution but might not help older patients since our informants do not even use apps to remember to take the medication. A simpler alternative could be a label on the distributed product indicating that it is an alternative to the original prescription.

For frail older patients with difficulties managing their medication, the opportunity for assistance from the municipalities’ home care is vital. Patients with suspected declining mental function risk losing their ability to manage medication and should be offered help.

### Timing and medication schedule

In this study, the timing of medication was important for patients and relatives with specific requests for more precise instructions about when medication should be taken to ensure optimum treatment and avoid interactions. Patients request a recommendation for a specific time, even if the medication only needs to be taken once a day. In Denmark, instructions for prescribed medication commonly state that the medication should be taken, for example, morning, noon, and evening. Extra information about timing could be provided in writing regarding when medication should be taken, e.g. every 8 h with a deviation of +/-2 h or combined after dialogue with the patient about specific, suitable times and added to the medication list. In addition, a graphic illustration of medication timing for polypharmacy patients may improve understanding. However, improving this factor should be tested in a rigorous research design to see if it can improve medication adherence.

### Medication reviews

Studies show that patients are often concerned about the side effects and interactions with other prescribed medications [37, 38]. Medication reviews conducted by physicians or pharmacists can reduce treatment problems and identify challenges related to the medication [39] but are time consuming and hence costly. In this study, many patients requested more opportunities for a medication review. Patients receive different information from healthcare professionals and package leaflets, resulting in fear of possible side effects or interactions. Patients lose confidence, and sometimes discontinue the prescription when they feel the medication is not thoroughly reviewed. The patients indicated that one healthcare professional should be responsible for reviewing and adjusting the patient’s medication. In Denmark, general practitioners can access tools to support a medication review. In addition, all patients can be referred to outpatient clinics specializing in optimizing medications, and older patients to geriatric outpatient clinics [40]. Increased utilization of these resources may improve the patients’ experiences and need to be studied more specifically.

Patients and relatives describe needing an overview of possible side effects and interactions. This could easily be built into an app assisting patients in remembering their medication. However, our FGI’s also show that the participants aged 72 or older chose not to use an app for medication management. A study from 2014 indicated promising results when an app was used for patients aged 65 and above [41]. However, the participants from this study prefer contact with healthcare professionals combined with printed medication lists. Younger generations may be expected to be more familiar with using cell phones and apps [42] and have the same need for information about their medication. Therefore, apps may be more useful in the coming years. An interactive collection of information in an app might satisfy many patients’ wishes. However, given how the interviewed patients were worried by the information in the prescription leaflets, the app must be developed to encourage individuals to take their medication as planned rather than scaring them into Intentional non-adherence.

#### Intentional non-adherence

Interestingly, some patients choose to discontinue medication based on information from package leaflets and wait until a future appointment with the physician to discuss the decision [33]. Similarly, some patients experienced side effects but kept taking the medication despite the discomfort until they could discuss it with a physician [33]. In another study [43], 11% of geriatric patients with chronic illnesses deliberately opted not to take a particular medication, due to side effects or financial limitations [43–45]. None of the participants in this study reported intentional non-compliance due to financial concerns. The Danish welfare state ensures that all older people receive a national pension and medication subsidies [34].

A systematic review of interventions to increase medication compliance showed that verbal and verbal/written information was the most effective [35]. The authors concluded that the effect was because patients suffered from cardiovascular diseases where non-compliance has life-threatening consequences [35, 36]. This is similar to findings from other studies [46]. However, the results of this study indicate that thorough information about their medication is important for patients, and it is an essential prerequisite for improving patients’ ability to medicate themselves. Thus, the prescribing physician should discuss the new medication’s side effects in relation to the information on the package leaflet so the patient is aware that the treatment is correct, even if the package leaflet warns against the medication with certain conditions.

### Unintentional non-adherence

The patients and relatives do not report significant problems in remembering to take their medication except when routines are interrupted or they are preoccupied, which is similar to other studies that reported that age itself is not a predictor of non-adherence [47]. However, forgetting to take medication is common [48]. Some of the participating patients reported using an alarm on the phone to remember to take their medications if preoccupied or interrupted.

#### Dose dispensing/pill organizers

While medication management is a practice that must be fitted into everyday life, it is also the subject of many considerations, concerns, and wishes among patients and relatives. The patients in this study who had tried dose dispensing in pill organizers packed by the home care team were satisfied with the service. Whilst there were no patients that had tried dose dispensing using blisters. Blister-paced dose dispensing also makes adjustments difficult, especially during a pre-packed period. Other studies also show that dose dispensing can make it difficult for the primary care sector to maintain an overview of the patient’s medication after discharge from hospital [38]. Another concern among healthcare professionals regarding dose dispensing is that it may be costly for patients if a medication change results in dose-dispensed medication being regarded as waste. This aspect was, however, not voiced by the participating patients.

#### Perspectives

While the medication lists greatly help the participating, well-functioning patients, patients request more guidance and knowledge about their medication. Our study indicates that this should include suggestions for when the medication can be taken, combined with intervals showing how much the patient can deviate from this schedule. Nurses also requested this knowledge, which may reduce the number of phone calls to the GPs. In addition, the medication list could be supplemented with a list of generic medications that can replace the prescribed medication helping the patient to maintain an overview of which generic medication can replace the prescribed medication. This solution could be created digitally as an app. The design and applicability of these elements should be examined in a rigorous future research design examining whether lists of side effects and interactions help the patient establish an overview and increase confidence in the medications and perhaps reduce patients’ desires for a medication review.

#### Strengths and limitations

Patients were interviewed after discharge from the hospital. However, they were asked if they would participate in the study during their hospitalization where they were informed verbally and provided with patient-oriented information material. After discharge, the patient was contacted by telephone by THM to arrange the FGI. Hence, the patients had the opportunity not to participate. Approaching the patients at the hospital may be a limitation if patients felt obliged to participate. However, the patients had many other opportunities to decline participation.

Bias may occur if the informants do not speak their minds and may always affect FGI’s. Hence the surroundings and meeting facilities may influence the participants, and the participants may have affected each other. Therefore, it is important to ensure good meeting facilities and a good dialogue, ensuring that different opinions and experiences are heard without judgments. The meeting rooms at the hospital were arranged as classic meeting rooms without hospital characteristics, and neither interview nor analysis shows that the informants were affected by the environment. One of the FGI’s comprised of a patient and relative as well as a GP, pharmacist, homecare nurse, and in the FGI the patient may have felt insecure and refrained from speaking out. However, the patient and the relative did not seem affected and spoke freely, and the atmosphere was friendly and relaxed. Their participation is considered to be a strength as they participated and enriched the discussion in the group. However, their participation may also have caused healthcare professionals to moderate their opinions of patients, treatments, or medications to avoid offending the patient and relative. THM is a trained researcher in qualitative methods and ensured that the participants had good opportunities to speak their minds by asking open-ended questions and additional non-biased clarifying questions. THM ensured that all voices were heard and all experiences and opinions were accepted and debated properly. As a sociologist, THM had no prior expectations regarding patient medications and the possible problems facing older patients after discharge from hospital or knowledge of medication challenges forhealthcare professionals.

A strength of this study is that patients and relatives managed medication daily and were mentally well functioning. They answered relevantly and clearly and participated in the dialogue with the interviewer and the other informants expressing experiences and feelings. A limitation of the study is that frail senior citizens may be underrepresented,and patients taking no particular interest in their medication might be expected to decline participation in the FGI’s. However, the participants had a high degree of knowledge about their conditions and were willing to discuss the central problems of polypharmacy. Patients are probably the best informants to highlight the factors preoccupying this target group. Another limitation is that all participating patients were Danish, so the results may not represent issues relevant to older patients of other ethnic backgrounds.

FGI’s allow participants to challenge, elaborate, and clarify questions illuminating the informants’ opinions. Likewise, FGI’s, including patients and healthcare professionals, create a platform for listening to a unique dialogue. Including healthcare professionals enables the perspectives of weaker patients or patients with no particular interest in their medication to be included.

## Conclusion

Physicians must prioritize giving information regarding when medication should be taken, including an acceptable timespan deviation to reduce patient concern about potential medication interactions. Timing of the medication is also requested by hospital and homecare nurses,. Medication lists are helpful to well-functioning patients and may be supplemented by a list of generic medications that can replace the prescribed medication helping the patient to maintain an overview of the medication. However, information from different actors compared with information from package leaflets, may lead to intentional non-adherence. Therefore, patients often request a thorough medication review to ensure an overview of the medication, side effects, interactions, and timing. Future studies should address whether the patients may benefit from a list or an app including more information about these aspects. In addition, the design and applicability of these elements should be examined in a future rigorous research design which could explore whether lists of side effects and interactions help the patient establish an overview, increase confidence in the medications, and perhaps reduce patients’ desires for a medication review.

### Electronic supplementary material

Below is the link to the electronic supplementary material.

Supplementary Material 1

## Data Availability

The datasets are not publicly available due to regulations from The Danish Data Protection Agency but are available from the corresponding author on reasonable request.

## Abbreviations

FGI: Focus Group Interview
GP: General practitioner
SMR: an online Shared Medication Record that can be accessed by the patient and healthcare professionals across sectors. In Danish called Fælles Medicinkort (FMK)
