# Supplementary material for: Handling polypharmacy –a qualitative study using focus group interviews with older patients, their relatives, and healthcare professionals
Source: BMC Geriatr. 2023 Aug 8;23:477. doi: 10.1186/s12877-023-04131-6 (PMC10410867; doi:10.1186/s12877-023-04131-6)
Supplement: Supplementary file 1 — Supplementary Material 1 [file 12877_2023_4131_MOESM1_ESM.docx]

**Supplementary files:**

**Key elements, challenges and opportunities regarding** **older patients' medication at discharge from hospital - healthcare professionals' experiences.**

**Authors**

Thorbjørn Hougaard Mikkelsen 1,2,3

Jens Søndergaard 3

Niels Kristian Kjær 3

Jesper Bo Nielsen 3

Jesper Ryg 4,5

Lene Juel Kjeldsen 6

Christian Backer Mogensen 1,2

1 Emergency Department, Hospital Sønderjylland, Denmark. 2 Research Unit of Emergency Medicine, Department of Regional Health Research, University of Southern Denmark, Odense, Denmark. 3 Research Unit of General Practice, Department of Public Health, University of Southern Denmark, Odense, Denmark. 4 Department of Clinical Research, University of Southern Denmark, 5000 Odense, Denmark. 5 Department of Geriatric Medicine, Odense University Hospital, Odense, Denmark. 6 The hospital pharmacy research unit, Hospital Sønderjylland, Denmark.

**Corresponding author:**

Thorbjørn Hougaard Mikkelsen

Hospital Sønderjylland,

Kresten Philipsens vej 15, indgang F

6200 Aabenraa, Denmark.

[Thorbjorn.Hougaard.Mikkelsen@rsyd.dk](mailto:Thorbjorn.Hougaard.Mikkelsen@rsyd.dk)

**Supplementary file 1: Interview guides.**

**Translated from the original Danish language versions**

***Semi-structured Interview guide for FGI A***

***Interview guide FGI A Hospital staff***

**Introductory remarks:**

Thank you for coming today. My name is Thorbjørn Hougaard Mikkelsen and I am a sociologist with a Ph.D. from Aarhus University and affiliation with the Research Unit for General Practice at SDU. I also have connections to Sygehus Sønderjylland. The form in front of youis the declaration of consent, one section is for your own information and the other I would like you to complete and return to me. This interview will be recorded and is confidential and will only be used for research. Your participation is voluntary, and you can always withdraw consent. If you do this then your information will not be used in the research project.

This project aims to develop a method or solution to ensure that patients over 71 years old, with more than 4 types of medication receive the correct medication after discharge from hospital . The first step today will be to listen to the challenges you experience, either in relation to whether the patient takes the medicine as instructed or other knowledge you have in relation to medication during hospitalization and at discharge. In short, I would like to understand the challenges you are experiencing and discuss possible solutions.

In this process, we will also discuss these challenges with patients, relatives, hospital doctors, pharmacists, nurses and home care nurses and assistants. After we have interviewed all these groups we collate and develop a solution that works for all of you during a busy working day. The discussion today is confidential, only I and a few other researchers have access to these recordings for analysis purposes. in the results of this discussion will be used in scientific articles and for the development of new tools for the benefit of patients, pharmacists, doctors, nurses and SOSU assistants, so everyone can be onboard to ensure the patient receives the correct medicine.

So just speak freely, you are my chance to find out how we can this better. It would help me enormously if you only please speak one at the time.

Do you have any questions?

I start the dictaphone.

**Questions to support the interview**:

First, lets start with names department and position so it's easier for me to hear who's saying what when I replay the interview again.

Now let’s begin with a shot in the dark; what challenges have you experienced, in relation to medication for elderly patients recently discharged from hospital?

Hospitalization often results in many medication changes for patients.

What does that mean?

For the patient that they are going to take new, more or other medication?

For your partners in the primary sector?

When do you typically look up a patient's medication?

- At admission? During hospitalization?, at discharge? after discharge?
- When is there typically a reason for changes in the medication?
- When is there a typical cause for changes in the medication after discharge, as far as you know?
- How do you handle it?

What is your experience of patients own overview of the medication?

- Do you know how they manage their medication?
- Do you know if they have assistance? (and by whom?)
- How do they handle changes in the ir medication?

Are there challenges in getting patients to take their medication?

- - Describe these?
- - When do you typically become aware that a patient is not taking their medication correctly?
- - Why aren’t these medications taken correctly?
- - Describe some good experiences with solutions that work for patients?
- What do you think are the most important factors for a patient to take their medication correctly?

Can you describe your overview of an elderly patient's medication at discharge from hospital?

- Overview?

- Experience of complete control of medication?

- Interactions?

- What is missing?

- What problems do you experience and what are the causes?

- How is your overview of an older patient's medication within the first week after at discharge from hospital?

There can be many different players in relation to a patient's medication. What are your impressions in relation to access to correct knowledge and these players - is access to correct knowledge relevant to them?

Imagine an ideal world, where all your wishes comes true. In this world, how would you ensure optimal procedures to ensure correct medication when an elderly patients has a sector transition?

How would this help?

Is there more that needs to be done?

Who would this help?

If this solution existed, what would it make easier for you?

Could it be good if it could do something more?

Do you have a suggestion about how it would look?

Thankyou. I have no further questions.

But before I turn off the recording, I want to know if there is anything more that needs to be said about medication for the vulnerable elderly patients after discharge from hospital?

Thank you for your participation.

**Semi-structured Interview guide for FGI B and C**

***Interview guide: primary healthcare sector including nurses from general practice, homecare nurses, home care assistant and pharmacists.***

**Introductory remarks:**

Good morning and thank you for coming today. My name is Thorbjørn Hougaard Mikkelsen and I am a sociologist with a Ph.D. from Aarhus University and affiliation with the Research Unit for General Practice at SDU. I also have connections to Sygehus Sønderjylland. The form in front of youis the declaration of consent, one section is for your own information and the other I would like you to complete and return to me. This interview will be recorded and is confidential and will only be used for research. Your participation is voluntary, and you can always withdraw consent. If you do this then your information will not be used in the research project.

This project aims to develop a method or solution to ensure that patients over 71 years old, with more than 4 types of medication receive the correct medication after discharge from hospital . The first step today will be to listen to the challenges you experience, either in relation to whether the patient takes the medicine as instructed or other knowledge you have in relation to medication during hospitalization and at discharge. In short, I would like to understand the challenges you are experiencing and discuss possible solutions.

In this process, we will also discuss these challenges with patients, relatives, hospital doctors, pharmacists, nurses and home care nurses and assistants. After we have interviewed all these groups we collate and develop a solution that works for all of you during a busy working day. The discussion today is confidential, only I and a few other researchers have access to these recordings for analysis purposes. in the results of this discussion will be used in scientific articles and for the development of new tools for the benefit of patients, pharmacists, doctors, nurses and SOSU assistants, so everyone can be onboard to ensure the patient receives the correct medicine.

So just speak freely, you are my chance to find out how we can this better. It would help me enormously if you only please speak one at the time.

Do you have any questions?

I start the dictaphone.

**Questions to support the interview:**

First, lets start with names so it's easier for me to hear who's saying what when I replay the interview again.

Now let’s begin with a shot in the dark; what challenges have you experienced, in relation to medication for elderly patients recently discharged from hospital?

Hospitalization often results in many medication changes for patients.

What does that mean?

To you?

To your patient?

Can you describe your overview of an elderly patient's medication immediately after discharge from hospital?

- Overview?

- Experience of complete control of medication?

- Interactions?

- What is missing?

- What problems do you experience and what are the causes?

When do you typically look up a patient's medication?

- After discharge?

- After discharge, what time is typical for changes in medication?

What is your experience of patients own overview of the medication?

- Do you know how they manage their medication?

- Do you know if they have assistance? (and by whom?)

Are there challenges in getting patients to take their medication?

- Describe these?

- When do you typically become aware that a patient is not taking their medication correctly?

- Why aren’t these medications taken correctly?

- Describe some good experiences with solutions that work for patients?

What do you think are the most important factors for a patient to take their medication correctly?

There can be many different players in relation to a patient's medication. What are your impressions in relation to access to correct knowledge and these players - is access to correct knowledge relevant to them?

Imagine an ideal world, where all your wishes comes true. In this world, how would you ensure optimal procedures to ensure correct medication when an elderly patients has a sector transition?

- How would this help?

- Is there more that needs to be done?

- Who would this help?

If this solution existed, what would it make easier for you?

Could it be good if it could do something more?

Do you have a suggestion about how it would look?

Thankyou. I have no further questions.

But before I turn off the recording, I want to know if there is anything more that needs to be said about medication for the vulnerable elderly patients after discharge from hospital?

Thank you for your participation.

**Semi-structured Interview guide for FGI D**

***Interview guide General Practitioners***

**Introductory remarks:**

Good morning and thank you for coming today. My name is Thorbjørn Hougaard Mikkelsen and I am a sociologist with a Ph.D. from Aarhus University and affiliation with the Research Unit for General Practice at SDU. I also have connections to Sygehus Sønderjylland. The form in front of youis the declaration of consent, one section is for your own information and the other I would like you to complete and return to me. This interview will be recorded and is confidential and will only be used for research. Your participation is voluntary, and you can always withdraw consent. If you do this then your information will not be used in the research project.

This project aims to develop a method or solution to ensure that patients over 71 years old, with more than 4 types of medication receive the correct medication after discharge from hospital . The first step today will be to listen to the challenges you experience, either in relation to whether the patient takes the medicine as instructed or other knowledge you have in relation to medication during hospitalization and at discharge. In short, I would like to understand the challenges you are experiencing and discuss possible solutions.

In this process, we will also discuss these challenges with patients, relatives, hospital doctors, pharmacists, nurses and home care nurses and assistants. After we have interviewed all these groups we collate and develop a solution that works for all of you during a busy working day. The discussion today is confidential, only I and a few other researchers have access to these recordings for analysis purposes. in the results of this discussion will be used in scientific articles and for the development of new tools for the benefit of patients, pharmacists, doctors, nurses and SOSU assistants, so everyone can be onboard to ensure the patient receives the correct medicine.

So just speak freely, you are my chance to find out how we can this better. It would help me enormously if you only please speak one at the time.

Do you have any questions?

I start the dictaphone.

**Questions to support the interview:**

First, lets start with names so it's easier for me to hear who's saying what when I replay the interview again.

Now let’s begin with a shot in the dark; what challenges have you experienced, in relation to medication for elderly patients recently discharged from hospital?

Hospitalization often results in many medication changes for patients.

What does that mean?

To you?

To your patient?

Can you describe your overview of an elderly patient's medication immediately after discharge from hospital?

- Overview?

- Experience of complete control of medication?

- Interactions?

- What is missing?

- What problems do you experience and what are the causes?

When do you typically look up a patient's medication?

- After discharge?

- After discharge, what time is typical for changes in medication?

What is your experience of patients own overview of the medication?

- Do you know how they manage their medication?

- Do you know if they have assistance? (and by whom?)

Are there challenges in getting patients to take their medication?

- Describe these?

- When do you typically become aware that a patient is not taking their medication correctly?

- Why aren’t these medications taken correctly?

- Describe some good experiences with solutions that work for patients?

What do you think are the most important factors for a patient to take their medication correctly?

There can be many different players in relation to a patient's medication. What are your impressions in relation to access to correct knowledge and these players - is access to correct knowledge relevant to them?

Imagine an ideal world, where all your wishes comes true. In this world, how would you ensure optimal procedures to ensure correct medication when an elderly patients has a sector transition?

How would this help?

Is there more that needs to be done?

Who would this help?

If this solution existed, what would it make easier for you?

Could it be good if it could do something more?

Do you have a suggestion about how it would look?

Thankyou. I have no further questions.

But before I turn off the recording, I want to know if there is anything more that needs to be said about medication for the vulnerable elderly patients after discharge from hospital?

Thank you for your participation.

**Semi-structured Interview guide for FGI E**

***Interview guide: Mixed FGI with a GP, 1 homecare nurse, 1 hospital-employed pharmacist, 1 patient and 1 spouse.***

**Introductory remarks:**

Thank you for coming today. My name is Thorbjørn Hougaard Mikkelsen and I am a sociologist with a Ph.D. from Aarhus University and affiliation with the Research Unit for General Practice at SDU. I also have connections to Sygehus Sønderjylland. The form in front of youis the declaration of consent, one section is for your own information and the other I would like you to complete and return to me. This interview will be recorded and is confidential and will only be used for research. Your participation is voluntary, and you can always withdraw consent. If you do this then your information will not be used in the research project.

This project aims to develop a method or solution to ensure that patients over 71 years old, with more than 4 types of medication receive the correct medication after discharge from hospital . The first step today will be to listen to the challenges you experience, either in relation to whether the patient takes the medicine as instructed or other knowledge you have in relation to medication during hospitalization and at discharge. In short, I would like to understand the challenges you are experiencing and discuss possible solutions.

In this process, we will also discuss these challenges with patients, relatives, hospital doctors, pharmacists, nurses and home care nurses and assistants. After we have interviewed all these groups we collate and develop a solution that works for all of you during a busy working day. The discussion today is confidential, only I and a few other researchers have access to these recordings for analysis purposes. in the results of this discussion will be used in scientific articles and for the development of new tools for the benefit of patients, pharmacists, doctors, nurses and SOSU assistants, so everyone can be onboard to ensure the patient receives the correct medicine.

So just speak freely, you are my chance to find out how we can this better. It would help me enormously if you only please speak one at the time.

Do you have any questions?

I start the dictaphone.

**Questions to support the interview:**

First, lets start with names so it's easier for me to hear who's saying what when I replay the interview again.

Now let’s begin with a shot in the dark; what challenges have you experienced, in relation to medication for elderly patients recently discharged from hospital?

Hospitalization often results in many medication changes for patients.

What does that mean?

To you? (Asking all around the table)

Can you describe your overview of an elderly patient's medication immediately after discharge from hospital?

- Overview?

- Experience of complete control of medication?

- Interactions?

- What is missing?

- What problems do you experience and what are the causes?

When do you typically look up a patient's medication?

- After discharge?

- After discharge, what time is typical for changes in medication?

What is your experience of patients own overview of the medication?

- Do you know how they manage their medication?

- Do you know if they have assistance? (and by whom?)

Addressing the patients: Do you sometimes find it difficult to figure out how to take the medicine? Or is there something else that gets in the way, for example, that they are physically difficult to consume or have uncomfortable side effects?

Are there challenges in getting patients to take their medication?

- Describe these?

- When do you typically become aware that a patient is not taking their medication correctly?

- Why aren’t these medications taken correctly?

- Describe some good experiences with solutions that work for patients?

Addressing the patients: What do you think are the most important factors who may help you to take your medication correctly?

What do you think are the most important factors for a patient to take their medication correctly?

There can be many different players in relation to a patient's medication. What are your impressions in relation to access to correct knowledge and these players - is access to correct knowledge relevant to them?

Imagine an ideal world, where all your wishes comes true. In this world, how would you ensure optimal procedures to ensure correct medication when an elderly patients has a sector transition?

How would this help?

Is there more that needs to be done?

Who would this help?

If this solution existed, what would it make easier for you?

Could it be good if it could do something more?

Do you have a suggestion about how it would look?

Thankyou. I have no further questions.

But before I turn off the recording, I want to know if there is anything more that needs to be said about medication for the vulnerable elderly patients after discharge from hospital?

Thank you for your participation.

**Semi-structured Interview guide for FGI F,G and H**

***Interview guide: patients and patients and spouses.***

**Introductory remarks:**

Thank you very much for choosing to participate. My name is Thorbjørn Hougaard Mikkelsen. I will need you to fill out the declaration of consent form, one part is for you to take home, and the other needs to be filled out and returned to me. The interview will be recorded and as you can see is confidential and will only be used for research purposes. Your participation is voluntary, and you can always withdraw consent. If you do that, then we will not use your information in the research project. In addition, there is a form to ensure you receive payment for the travel allowance, which you can fill out and return to me.

This project aims to develop a method or solution to ensure that you and other patients receive the correct medication after discharge from a hospital - not too little or too much. The way to develop this is to listen to and understand the challenges patients, relatives, doctors, pharmacists, nurses, and home care nurses and assistants experience. Collating these challenges we can develop a solution that works for all professionals in a busy everyday life. The first step is to explore how you handle your medication everyday. There is no right or wrong answer, and potentially what you experience may be similar to others, even if you are the only one in the group to describe it. Therefore, I ask you to talk freely about your knowledge and experiences. The discussion today is confidential, only I and a few other researchers have access to these recordings for analysis purposes. The results of this discussion will be used in scientific articles and for the development of new tools for the benefit of patients, doctors, pharmacists, doctors, nurses, and SOSU assistants, so everyone can be on board to ensure you get the optimal medicine.

So just speak freely, you are my chance to find out how we can do this better.

Do you have any questions?

I start the dictaphone.

**Questions to support the interview:**

First, lets start with names so it's easier for me to hear who's saying what when I replay the interview again.

Now lets start with a round where you describe how you usually take your medicine. You do not have to mention the kind of medicine or what condition it’s prescribed for – that’s your own decision.

- How many types of medicine do you take? How often; morning, noon, and evening?

- How do you remember to take the medication?

- Does anyone or anything help you remember? How?

Personally, I find it difficult to remember to take medication.

Have you ever forgotten to take your medicine?

- Why?

- What could be done to help you remember your medication in these situations?

How do you make sure you remember to take your medicine? Do you have a specific plan, bag, or box or anything else that helps?

Is there anything else that is challenging for you when you take your medicine? (Remember, find, take?)

Sometimes the medication can be changed. Can you describe what happens when the medicine is altered? (e.g. type of medicine, dose, interaction at the pharmacy)

Personally, it is most difficult for me to remember to take medicine when I have been given a new type of medicine

- Is this also difficult for you?

- What do you do when you have changes in your medicine?

- How do you remember the changes? Do you have any tricks that work for you?

You have now told me about how you remember to take your medicine and when it is difficult to remember to take it: Do you have any other ideas of what to do to remember your medicine? (write lists, an alarm? Other ideas during ourdiscussion?)

If you invented "something" to help remember your medicine, what would that be?

How would it help?

Is there other things it needs to be able to do?

Do you have a suggestion about what it could look like?

Who would it help? You? Others?

If you had this solution, what becomes easier for you?

Could it be good if it could do more?

Thankyou. I have no further questions.

However, before I turn off the recording, I want to know if there is anything else that can make the medication process easier for you?

Thank you for your participation.
